# Supplementary material for: Single strain control of microbial consortia
Source: Nat Commun. 2021 Mar 30;12:1977. doi: 10.1038/s41467-021-22240-x (PMC8010080; doi:10.1038/s41467-021-22240-x)
Supplement: Supplementary file 1 — Supplementary Information [file 41467_2021_22240_MOESM1_ESM.pdf]

Supplementary Information: Single strain control of microbial consortia

Alex J.H. Fedorec[1], Behzad D. Karkaria[1], Michael Sulu[2], and Chris P Barnes*[1,3]

[1]Department of Cell and Developmental Biology, University College London, London, WC1E 6BT, UK
[2]Department of Biochemical Engineering, University College London, London, WC1E 6BT, UK
[3]UCL Genetics Institute, University College London, London, WC1E 6BT, UK

1 Simple Model of Competition with Bacteriocins in Chemostat

We have developed a simple model of competition within a chemostat environment and added the ability of the engineered strain to produce a bacteriocin that can kill a competitor

$$
\begin{aligned}
\frac{dN_E}{dt} &= (\mu_E - D)N_E \\
\frac{dN_C}{dt} &= (\mu_C - D - k_\omega\, B)N_C \\
\frac{dS}{dt} &= D(S_0 - S) - \frac{\mu_E N_E}{\gamma_E} - \frac{\mu_C N_C}{\gamma_C} \\
\frac{dB}{dt} &= k_B N_E - DB
\end{aligned}
$$

where N_E is the engineered population, N_C is the competitor population, S is the common growth substrate and B is the bacteriocin and the parameters are as defined in Table 1. The growth rates are given by Monod growth functions

$$
\begin{aligned}
\mu_E &= \frac{\mu_{E_{\max}} S}{K_E + S} \\
\mu_C &= \frac{\mu_{C_{\max}} S}{K_C + S}
\end{aligned}
$$

The model is extended to include quorum molecule control of bacteriocin expression by the addition of a quorum molecule species, Q

$$
\frac{dQ}{dt} = k_Q N_E - DQ
$$

*Corresponding author: christopher.barnes@ucl.ac.uk

Table 1: Model parameters

Parameter	Description	Unit
D	Dilution rate	h^{-1}
$\mu_{E_{\max}}$	Maximum growth rate of engineered strain	h^{-1}
$\mu_{C_{\max}}$	Maximum growth rate of competitor strain	h^{-1}
S_0	Reservoir substrate concentration	g L^{-1}
γ_E	Yield coefficient of engineered strain	cell g^{-1}
γ_C	Yield coefficient of competitor strain	cell g^{-1}
K_E	Concentration of substrate at which engineered strain growth is half-maximal	g L^{-1}
K_C	Concentration of substrate at which competitor strain growth is half-maximal	g L^{-1}
k_ω	Death rate of competitor cells from bacteriocin	M^{-1} h^{-1}
k_B	Expression rate of bacteriocin from engineered strain	mol $cell^{-1}$ h^{-1}
k_Q	Expression rate of quorum molecule from engineered strain	mol $cell^{-1}$ h^{-1}
$k_{B_{max}}$	Maximal expression rate of bacteriocin from engineered strain	mol $cell^{-1}$ h^{-1}
$k_{B_{min}}$	Minimal expression rate of bacteriocin from engineered strain	mol $cell^{-1}$ h^{-1}
K_Q	Concentration of Q at which bacteriocin expression is half-maximal	M
n	Cooperativity of bacteriocin repression by Q	

and making the production rate of the bacteriocin k_B dependent on Q

$$k_B \quad = \quad (k_{B_{\max}} - k_{B_{\min}}) \frac{K_Q^n}{K_Q^n + Q^n} + k_{B_{\min}}$$

These models were simulated in Python using the SciPy [1] odeint function. The introduction of the quorum molecule makes the system of equations too complex to solve analytically. Instead, for each set of parameters, we simulate the system for 5000 hours and take the species at the final timepoint as the steady state values.

2 Model space exploration equations

The space of all possible models was explored using a method we have previously described [2]. The complete system of equations used for our model consist of ordinary differential equations describing the time evolution of each species within the system

$$
\begin{aligned}
\frac{dN_E}{dt} &= N_E(\mu_E - \eta_E - \omega_E - D) \\
\frac{dN_C}{dt} &= N_C(\mu_C - \omega_C - D) \\
\frac{dS}{dt} &= D(S_{0_j} - S_j) - \frac{N_E\mu_E}{\gamma} - \frac{N_C\mu_C}{\gamma} \\
\frac{dQ}{dt} &= k_Q N_E - DQ \\
\frac{dB}{dt} &= k_B(Q)N_E - DB \\
\frac{dI}{dt} &= k_I(Q)N_E - I\frac{\mu_E}{2} \\
\frac{dT}{dt} &= k_T(Q)N_E - T\frac{\mu_E}{2} - \alpha(T,V) \\
\frac{dV}{dt} &= k_V(Q)N_E - V\frac{\mu_E}{2} - \alpha(T,V)
\end{aligned}
$$

$$
\begin{aligned}
\omega_E &= \begin{cases} 0 & \text{if } I \text{ constitutive,} \\ \omega_{\max}\frac{B^{n_\omega}}{K_\omega^{n_\omega}+B^{n_\omega}} \cdot \frac{K_I^{n_I}}{K_I^{n_I}+I^{n_I}} & \text{else} \end{cases} \\
\omega_C &= \omega_{\max}\frac{B^{n_\omega}}{K_\omega^{n_\omega}+B^{n_\omega}} \\
\eta &= \eta_{\max}\frac{T^{n_T}}{K_\eta^{n_\eta}+T^{n_\eta}} \\
\mu_E &= \mu_{E_{\max}}\frac{S}{K_E+S} \\
\mu_C &= \mu_{C_{\max}}\frac{S}{K_C+S}
\end{aligned}
$$

$$
k_B(Q) = \begin{cases} k_{B_{\max}}\frac{Q^{n_B}}{K_B^{n_B}+Q^{n_B}} & \text{if } B \text{ induced,} \\ k_{B_{\max}}\frac{K_B^{n_B}}{K_B^{n_B}+Q^{n_B}} & \text{if } B \text{ repressed,} \\ k_{B_{\max}} & \text{if } B \text{ constitutive,} \\ 0, & \text{if } B \text{ not expressed,} \end{cases}
\quad
k_T(Q) = \begin{cases} k_{T_{\max}}\frac{Q^{n_T}}{K_T^{n_T}+Q^{n_T}} & \text{if } T \text{ induced,} \\ k_{T_{\max}}\frac{K_T^{n_T}}{K_T^{n_T}+Q^{n_T}} & \text{if } T \text{ repressed,} \\ k_{T_{\max}} & \text{if } T \text{ constitutive,} \\ 0 & \text{if } T \text{ not expressed,} \end{cases}
$$

$$
k_V(Q) = \begin{cases} k_{V_{\max}}\frac{Q^{n_V}}{K_V^{n_V}+Q^{n_V}} & \text{if } V \text{ induced,} \\ k_{V_{\max}}\frac{K_V^{n_V}}{K_V^{n_V}+Q^{n_V}} & \text{if } V \text{ repressed,} \\ 0 & \text{if } V \text{ not expressed,} \end{cases}
\quad
k_I(Q) = \begin{cases} k_{I_{\max}}\frac{Q^{n_V}}{K_I^{n_I}+Q^{n_I}} & \text{if } I \text{ induced,} \\ k_{I_{\max}}\frac{K_I^{n_T}}{K_I^{n_I}+Q^{n_I}} & \text{if } I \text{ repressed,} \\ k_{I_{\max}} & \text{if } I \text{ constitutive,} \\ 0 & \text{if } I \text{ not expressed,} \end{cases}
$$

Growth Equations

The change in the population of the engineered strain (N_E) is determined by the concentration of nutrient (S), bacteriocin (B), immunity protein (I), toxin proteins (T). The change in population of the competitor strain

(N_C) is dependent upon the concentration of substrate and bacteriocin

$$\mu_E = \mu_{E_{\max}} \frac{S}{K_E + S}$$
$$\mu_C = \mu_{C_{\max}} \frac{S}{K_C + S}$$

Detrimental effect of bacteriocin B and the protection provided by immunity I are described using Hill equations, whereby the negative effect of B is repressed by I. If the engineered strain is constitutively expressing I, immunity is constant, therefore, ω_E goes to 0

$$\omega_E = \begin{cases} 0 & \text{if I constitutive,} \\ \omega_{\max} \frac{B^{n_\omega}}{K_\omega^{n_\omega} + B^{n_\omega}} \cdot \frac{K_I^{n_I}}{K_I^{n_I} + I^{n_I}} & \text{else} \end{cases}$$
$$\omega_C = \omega_{\max} \frac{B^{n_\omega}}{K_\omega^{n_\omega} + B^{n_\omega}}$$

The detrimental effect of toxin T is also described as a Hill function

$$\eta = \eta_{\max} \frac{T^{n_T}}{K_\eta^{n_\eta} + T^{n_\eta}}$$

Bringing these functions together we can assemble differential equations describing the change in the population of each strain. The positive term being the growth rate, and negative terms being dilution rate, killing by bacteriocin and killing by toxin

$$\frac{dN_E}{dt} = N_E(\mu_E - \eta - \omega_E - D)$$
$$\frac{dN_C}{dt} = N_C(\mu_C - \omega_C - D)$$

Nutrient species

The change in the concentration of substrate S is dependent on consumption by N_E and N_C, and dilution with fresh media. N_E and N_C consume S with a yield constant, γ. The dilution term describes the continuous replenishment of nutrient with the stock nutrient (S_0)

$$S = D(S_0 - S) - N_E \frac{\mu_E}{\gamma} - N_C \frac{\mu_C}{\gamma}$$

Expressed species

Quorum molecule, Q, is expressed at a constant rate by the engineered strain

$$\frac{dQ}{dt} = k_Q N_E - DQ$$

The change in concentration of bacteriocin B is effected by expression from strains in the system and dilution by the chemostat environment. The form taken by $k_B(Q)$ is dependent on the method of regulation, which varies between models

$$\frac{dB}{dt} = k_B(Q)N_E - DB, \qquad k_B(Q) = \begin{cases} k_{B_{\max}} \frac{Q^{n_B}}{K_B^{n_B}+Q^{n_B}} & \text{if } B \text{ induced,} \\ k_{B_{\max}} \frac{K_B^{n_B}}{K_B^{n_B}+Q^{n_B}} & \text{if } B \text{ repressed,} \\ k_{B_{\max}} & \text{if } B \text{ constitutive,} \\ 0, & \text{if } B \text{ not expressed,} \end{cases}$$

Toxin (T), antitoxin (V) and immunity (I) species are all intracellular. The environmental dilution term is replaced with an intracellular dilution term derived from the rate of cell division of the engineered strain, $\frac{\mu_E}{2}$. Toxin and antitoxin species sequester one another, this is modelled as a linear relationship based on the concentration of the two species. Each species has different modes of expression which varies between the models. Constitutive expression of antitoxin V gives complete protection from T, we therefore discard these models (see Model space definition). Constitutive expression of I is reflected by changing ω_E to 0

$$\frac{dI}{dt} = k_I(Q)N_E - I\frac{\mu_E}{2}, \qquad k_I(Q) = \begin{cases} k_{I_{\max}} \frac{Q^{n_V}}{K_I^{n_I}+Q^{n_I}} & \text{if } I \text{ induced,} \\ k_{I_{\max}} \frac{K_I^{n_T}}{K_I^{n_I}+Q^{n_I}} & \text{if } I \text{ repressed,} \\ 0 & \text{if } I \text{ not expressed,} \end{cases}$$

$$\frac{dT}{dt} = k_T(Q)N_E - T\frac{\mu_E}{2} - \alpha(T,V), \qquad k_T(Q) = \begin{cases} k_{T_{\max}} \frac{Q^{n_T}}{K_T^{n_T}+Q^{n_T}} & \text{if } T \text{ induced,} \\ k_{T_{\max}} \frac{K_T^{n_T}}{K_T^{n_T}+Q^{n_T}} & \text{if } T \text{ repressed,} \\ k_{T_{\max}} & \text{if } T \text{ constitutive,} \\ 0 & \text{if } T \text{ not expressed,} \end{cases}$$

$$\frac{dV}{dt} = k_V(Q)N_E - V\frac{\mu_E}{2} - \alpha(T,V), \qquad k_V(Q) = \begin{cases} k_{V_{\max}} \frac{Q^{n_V}}{K_V^{n_V}+Q^{n_V}} & \text{if } V \text{ induced,} \\ k_{V_{\max}} \frac{K_V^{n_V}}{K_V^{n_V}+Q^{n_V}} & \text{if } V \text{ repressed,} \\ 0 & \text{if } V \text{ not expressed,} \end{cases}$$

$$\alpha(T,V) = k_{\text{ann}} \cdot T \cdot V$$

Example system - Figure 9 D iv

The engineered strain constitutively expresses Q. B is repressed by Q

$$\begin{aligned}
\frac{dN_E}{dt} &= N_E(\mu_E - D) \\
\frac{dN_C}{dt} &= N_C(\mu_C - \omega_C - D) \\
\frac{dS}{dt} &= D(S_{0_j} - S_j) - \frac{N_E\mu_E}{\gamma} - \frac{N_C\mu_C}{\gamma} \\
\frac{dQ_y}{dt} &= k_Q N_E - DQ \\
\frac{dB}{dt} &= \frac{K_B^{n_B}}{K_B^{n_B} + Q^{n_B}} N_E - DB
\end{aligned}$$

Model space definition

Species B, I and T can be expressed constitutively, induced, repressed or not expressed. Q can be either expressed constitutively or not expressed. V can be induced, repressed or not expressed. We represent expression options using integers. 0 constitutive, 1 induced, 2 repressed, 3 not expressed. We can define the options for each expressed species as such

$$B = 0 \text{ or } 1 \text{ or } 2 \text{ or } 3$$

$$I = 0 \text{ or } 1 \text{ or } 2 \text{ or } 3$$

$$T = 0 \text{ or } 1 \text{ or } 2 \text{ or } 3$$

$$V = 1 \text{ or } 2 \text{ or } 3$$

$$Q = 0 \text{ or } 3$$

We restrict the engineered strain to express up to one of each species. Let P_c be the number of unique part combinations, choosing one expression option for the engineered strain in each model

$$P_C = \binom{B}{1} \times \binom{I}{1} \times \binom{T}{1} \times \binom{V}{1} \times \binom{Q}{1}$$

We use a series of rules to remove illegal and redundant models from the model space. C_1 is a set of illegal models where if any of B, I, T or V are regulated by Q, while Q is not expressed. C_2 is a set of illegal models where I is expressed, and B is not expressed. C_3 is a set of illegal models where V is expressed and T is not expressed. C_4 is a set of illegal models where Q is expressed and none of B, I, T or V are regulated by Q.

Legal part combinations are taken by subtracting illegal combinations from the total unique part combinations

$$C_1 : (B \text{ or } I \text{ or } T \text{ or } V \in \{1, 2\} \text{ and } Q = 0)$$

$$C_2 : (I \in \{0, 1, 2\} \text{ and } B = 3)$$

$$C_3 : (V \in \{1, 2\} \text{ and } T = 3)$$

$$C_4 : (Q = 0 \text{ and } (B \ \& \ I \ \& \ T \ \& \ V) \notin \{1, 2\})$$

P_L is a set containing all the different ways in which the engineered strain can be engineered

$$P_L = P_C - (C_1 \cup C_2 \cup C_3 \cup C_4)$$

Table 2: Model priors

Parameter	Description	Lower bound	Upper bound	Prior distribution	Unit
D	Dilution rate	0.01	0.5	Uniform	h^{-1}
$\mu_{E_{\max}}$	Maximum growth rate of engineered strain	0.4	3	Uniform	h^{-1}
$\mu_{C_{\max}}$	Maximum growth rate of competitor strain	0.4	3	Uniform	h^{-1}
γ	Yield coefficient	$1e^{12}$	$1e^{12}$	Constant	cell g^{-1}
S_0	Concentration of S in input media	4	4	Constant	g L^{-1}
$\omega_{\max}$	Maximal death rate due to bacteriocin	0.5	2	Uniform	h^{-1}
$\eta_{\max}$	Maximal death rate due to bacteriocin	0.5	2	Uniform	h^{-1}
K_ω	Concentration of B at which killing rate is half-maximal	$1e^{-12}$	$1e^{-7}$	Uniform	M^{-1}
n_ω	Cooperativity coefficient for detrimental effect of bacteriocin	1	2	Uniform	
K_E	Concentration of S at which growth of engineered strain is half-maximal	2	2	Constant	gL^{-1}
K_C	Concentration of S at which growth of engineered strain is half-maximal	2	2	Constant	gL^{-1}
K_B	Concentration of Q at which expression of bacteriocin is half-maximal	$1e^{-9}$	$1e^{-7}$	Log uniform	M
K_I	Concentration of Q at which expression of immunity is half-maximal	$1e^{-9}$	$1e^{-7}$	Log uniform	M
K_T	Concentration of Q at which expression of toxin is half-maximal	$1e^{-9}$	$1e^{-7}$	Log uniform	M
K_V	Concentration of Q at which expression of anti-toxin is half-maximal	$1e^{-9}$	$1e^{-7}$	Log uniform	M
K_η	Concentration of T at which repression of growth is half-maximal	$1e^{-21}$	$1e^{-16}$	Log uniform	M
n_η	Cooperativity coefficient for detrimental effect of toxin	1	2	Uniform	$M^{-1}h^{-1}$
$k_{B_{\max}}$	Maximum expression rate of bacteriocin	$1e^{-21}$	$1e^{-19}$	Log uniform	$M^{-1}h^{-1}$
$k_{I_{\max}}$	Maximum expression rate of immunity	$1e^{-21}$	$1e^{-19}$	Log uniform	$M^{-1}h^{-1}$
$k_{T_{\max}}$	Maximum expression rate of toxin	$1e^{-21}$	$1e^{-19}$	Log uniform	$M^{-1}h^{-1}$
$k_{V_{\max}}$	Maximum expression rate of anti-toxin	$1e^{-21}$	$1e^{-19}$	Log uniform	$M^{-1}h^{-1}$
n_B	Cooperativity coefficient for expression of bacteriocin	2	2	Constant	
n_I	Cooperativity coefficient for expression of immunity	2	2	Constant	
n_T	Cooperativity coefficient for expression of toxin	2	2	Constant	
n_V	Cooperativity coefficient for expression of anti-toxin	2	2	Constant	
k_Q	Production rate of Q	$1e^{-20}$	$1e^{-19}$	Log uniform	$M^{-1}h^{-1}$
k_{ann}	Rate of toxin anti-toxin annihilation	30	30	Constant	$M^{-1}h^{-1}$

3 Model selection with ABC SMC

Bayesian inference

Let $\theta \in \Theta$ be a parameter vector with a prior $\pi(\theta)$. Given an objective of x_0, where x_0 exists in the solution space, $x_0 \in \mathcal{D}$. We define the likelihood function for the objective behaviour as $f(x_0|\theta)$

$$\pi(\theta|x_0) = \frac{f(x_0|\theta)\pi(\theta)}{\pi(x_0)}$$

We can rewrite $\pi(x_0)$ where a and b represent the lower and upper bounds of the parameter value

$$\pi(x_0) = \int_a^b f(x_0, \theta)d\theta = \int_a^b f(x_0|\theta)\pi(\theta)d\theta$$

The posterior distribution represents the parameter distribution that gives rise to the objective

$$\pi(\theta|x_0) = \frac{f(x_0|\theta)\pi(\theta)}{\int_a^b f(x_0|\theta)\pi(\theta)d\theta}$$

Let m be a model from vector of competing models, M, such that $m \in M = \{m_1, m_2...m_q\}$. Each model has its own parameter space, allowing us to define a joint space, $(m, \theta) \in M \times \Theta_M$. We can write Bayes' theorem in the context of a model space

$$\pi(m|x_0) = \frac{f(x_0|m)\pi(m)}{\int_M f(x_0|m')\pi(m')dm'}$$

Since the M is discrete, we can rewrite this

$$\pi(m|x_0) = \frac{f(x_0|m)\pi(m)}{\sum_M f(x_0|m')\pi(m')}$$

The marginal likelihood of the model, $f(x_0|m)$, is the expectation of the likelihood function taken over the model parameter prior distribution

$$f(x_0|m) = \int_\Theta \pi(\theta|m)f(x_0|\theta, m)d\theta$$

Approximate Bayesian computation

Writing the likelihood function, $f(x_0|\theta)$, in terms of complex summary statistics can be difficult. We bypass this and approximate the posterior by generating data from a model. We can sample a parameter vector from the prior, $\theta^* \sim \pi(\theta)$, which is simulated to yield a data vector, x^*. This can be written as a conditional, $x^* \sim f(x|\theta^*)$, which also gives the joint density, $\pi(\theta, x)$. In order to obtain the posterior distribution that satisfies our objective behaviour, x_0, we apply a conditional to define whether a generated data vector, x^* belongs to the objective x_0.

If $x = x_0$

$$\pi(\theta|x, x_0) = \frac{\pi(\theta)f(x|\theta)}{\pi(\theta)f(x|\theta)dxd\theta}$$

Else

$$\pi(\theta|x, x_0) = 0$$

Let $\rho(x, x_0)$ be a distance function that compares a simulation to the objective. Using distance thresholds (ϵ), we can define values below which the distance is acceptably small. We can redefine $\pi(\theta|x, x_0)$ in the context of thresholds to obtain an approximation of the posterior:

If $\rho(x, x_0) < \epsilon$

$$\pi_\epsilon(\theta|x, x_0) = \frac{\pi(\theta)f(x|\theta)}{\pi(\theta)f(x|\theta)dxd\theta}$$

Else

$$\pi_\epsilon(\theta|x, x_0) = 0$$

The smaller ϵ is and the larger the number of simulations conducted, the more accurate the representation of the true posterior will be

$$\pi(\theta^*|\rho(x^*, x_0)) \leq \epsilon \approx \pi(\theta|x_0)$$

ABC rejection algorithm

The most basic ABC algorithm is the ABC rejection algorithm. Let ϵ be the distance threshold defining the necessary level of agreement between the objective, x_0, and a given simulation, x^* (Algorithm 1). Particles refer to a model and vector of parameters necessary to simulate the model. The ABC rejection algorithm repeatedly samples particles from the prior distribution, $\pi(m, \theta)$, generating data by simulation and testing for its representation of an objective data. Particles that are able to reproduce the objective data are accepted. By repeating this process we build a population of accepted particles that represent the posterior distribution for the objective.

Model selection with ABC SMC

In this paper we use a variant of ABC, ABC Sequential Monte Carlo (ABC SMC) [3]. ABC SMC evolves the prior distribution through a series of intermediate distributions, each of which more closely resembles

Algorithm 1: ABC rejection algorithm

Set particle indicator $i = 0$
while $i < N$ **do**
  Sample model m, from model space prior, M
  Sample parameter θ^*, from prior distribution $\pi(\theta)$
  Simulate model, $f(x|\theta^*)$, giving simulation data, x^*
  Calculate distance between simulation data and objective, $d(x^*, x)$
  if $d(x^*, x) \leq \epsilon$ **then**
    Set $\theta_t^i = \theta^*$
    i = i + 1
  else
    Reject θ^*
end while
Posterior distribution generated from accepted particles, $\pi(\theta^*|d(x^*, x)) \leq \epsilon$

the posterior distribution than the previous (Algorithm 2). The distance threshold (ϵ) is decreased between distributions, moving the acceptance criteria closer to the objective. The gradual evolution reduces the tendency of becoming focused on local areas of minimal distance. In these experiments we use a component-wise Gaussian perturbation kernel. This produces a random walk from a particle of the previous population to a particle of the next population

$$K_t(\theta|\theta^*) = \mathcal{N}(\theta^*, 2x)$$

Where, x, is the variance of the previous population

$$x = \sigma(\theta_{t-1})^2$$

Defining stable steady state objective

We define the stable steady state objective with three summary statistics, where x is the time series data of a strain (N_E or N_C). Each distance function has been chosen to filter out undesirable behaviours leaving the remaining desired stable steady state behaviour. d_1 is the final gradient of x, capturing the fundamental feature of stable steady state

$$d_1(x) = |\Delta x(t-1)|$$

d_2 is the standard deviation of the signal, this filters out simulations which are oscillating

$$d_2(x) = \sigma(x)$$

d_3 is the reciprocal the final value of the simulation, this allows us to define a minimum population threshold

$$d_3(x) = \frac{1}{x(t-1)}$$

Algorithm 2: Model selection with ABC SMC

1 Set population indicator, $t = 0$
2 Set initial epsilon, $\epsilon_t = \inf$
3 Set final epsilon, $\epsilon_T = [x, y, z]$
4 Set particle indicator, $i = 0$
5 **if** $t = 0$ **then**
6   Sample m^* from $\pi(m)$
7   Sample θ^{**} from $\pi(\theta(m^*))$
8 **else if** $t > 0$ **then**
9   Sample particle θ^* from previous population $\{\theta(m^*)^i_{t-1}\}$ with weights $w(m^*)_{t-1}$
10   Perturb θ^* to obtain $\theta^{**} \sim K_t(\theta|\theta^*)$
11   **if** $\pi(\theta^{**}) = 0$ **then**
12   **go to** 5
13
14 Simulate, $x^* \sim f(x|\theta^{**}, m^*)$
15   **if** $d(x^*, x_0) > \epsilon_t$ **then**
16   **go to** 5
17
18 Set $m^i_t = m^*$
19 Set $\theta^i_t = \theta^{**}$
20 Calculate particle weight, w^i_t
21   **if** $t = 0$ **then**
22         $w^i_t = 1$
23   **else**
24         $w^i_t = \frac{\pi(\theta^{**})}{\sum_{j=1}^{N} w^j_{t-1} K_t(\theta^i_{t-1}|\theta^{**})}$
25   **if** $i < N$ **then**
26   Set $i = i + 1$
27   **go to** 5
28
29 Normalise weights for every m.
30 **if** $\epsilon_t \neq \epsilon_T$ **then**
31   Update population number, $t = t + 1$
32   Update ϵ according to accepted particle distances, $\epsilon_t = f_\epsilon()$
33   **go to** 5

Using these distances we can define the final conditional ϵ_F. The final threshold values we use are $\epsilon_F = \{500, 25000, 1e^{-10}\}$

$$d_1 < \epsilon_{F_1}$$

$$d_2 < \epsilon_{F_2}$$

$$d_3 < \epsilon_{F_3}$$

Auto ϵ generation

The next ϵ for each new population is generated based on the accepted particle distances from previous population until we reach the final ϵ. We calculate distances for each species we are fitting to the objective (N_E and N_C). Let X be time series data of a simulated particle and $\hat{X}$ contain all accepted particles in a population

$$X \in \hat{X}$$

From each simulation we can take the time series data of the species to be fit, X_{N_E} and X_{N_C}

$$X_{N_E} \in X$$

$$X_{N_C} \in X$$

These time series data are used to calculate the distances of the particle from the objective. We couple the distances of the two species being fit by taking the maximum of the two for each distance

$$V = \{X \in \hat{X} \mid \max(\{d_i(X_{N_E}), d_i(X_{N_C})\})\}$$

We set α as a proportion of the distances we should to generate the next ϵ. Generally speaking, the smaller α is the faster we will progress to ϵ_F

$$n = [\text{length}(\hat{X})/\alpha]$$

Let S be the n-th smallest distance, where $S \in V$. Allowing us to set the threshold for the next population, ϵ_{t+1}

$$\epsilon_{t+1} = S$$

4 Supplementary Figures & Tables

Table 3: Plasmids used in this work.

Plasmid	Description	Source
pSEVA281	pUC ori, KnR, MCS	SEVA collection
pSEVA637	BBR1 ori, GmR, GFP	SEVA collection
pBAD-mTagBFP2	BR322 ori, ApR, araC ← ParaBAD → mTagBFP2	[4]
pTD103luxI_sfGFP	ColE1 ori, KnR, luxR ← PLux → sfGFP-LAA, luxR ← PLux → luxI-LAA	[5]
pKDL071	ColE1 ori, KnR, GFPmut3b ← PLtetO-1, lacI ← PLtetO-1, Ptrc → tetR, Ptrc → mCherry	[6]
pMPES:V	p15A ori, CmR, ProTeOn → cvaC + cvi, mccV*	[7]
pMPES_AF01	p15A ori, CmR, PLtetO-1 → GFPmut3b + cvaC + cvi, mccV*	this work
pMPES_AF01d	pMPES_AF01 with a 2 bp deletion at the cvaC start codon	this work
p28_AF02	pUC ori, KnR, luxR ← PLux → sfGFP-LAA + tetR	this work
p28_AF03	pUC ori, KnR, araC ← ParaBAD → mTagBFP2 + luxI-LAA	this work
p28_AF04	pUC ori, KnR, AF02, AF03	this work
p23_AF04	p28_AF04 with BBR1 ori	this work
p27_AF04	p28_AF04 with SC101 ori	this work
p29_AF04	p28_AF04 with BR322 ori	this work
p28_AF041	p28_AF04 with GFP removed	this work
p23_AF041	p28_AF041 with BBR1 ori	this work
p63_AF041	p23_AF041 with Gentamicin resistance	this work
p63_AF043	p63_AF041 with constitutive mCherry	this work
pTNS1	Tn7 transposase expression, R6K ori, ApR	[8]
pTn7-M-Pem7_CFP	R6K ori, KnR, Pem7→CFP + GmR transposable cassette	SEVA collection

Table 4: Bacterial strains used in this work.

Strain	Description	Source
E. coli NEB 5-alpha		New England BioLabs
E. coli JW3910	Keio collection methionine auxotroph [9]	Horizon Discovery
E. coli MG1655		Prof. John Ward (UCL)
EcM-Gm-CFP	*E. coli* MG1655 with chromosomal Gentamicin resistance and CFP	this work

Table 5: Primers used for plasmid construction. Underlined nucleotides indicate annealing region, uppercase nucleotides indicate restriction enzyme recognition site.

Name	Sequence
P.SpeI.LuxI.R	ggctcgACTAGTagcacgcgtttacgctg
P.LuxI.F	aggatcgtacaggtttacgcaag
P.ApaI.GFP.R	cagctgGGGCCCtaagcttttacgctgcaagggc
P.XmaI.luxR.F	cgatcaCCCGGGcacctcgagttaatttttaaagtatgggc
P.ApaI.TetR.F	gtgactGGGCCCcggcgaagctagggac
P.SbfI.TetR.R	gtcaatCCTGCAGGcagtccgagctctcaagac
P.delGFP.R	gcactaGGGCCCattcgactataacaaaccattttcttgc
P.delGFP.F	gcatggatgagctctacaaagc
P.SEVA.ORI.R	tgttgtttgtcggtgaacgc
P.SEVA.ORI.F	cggtgctcaacgggaatc
P.SEVA.CARGO.R	cagatggagttctgaggtcatt
P.XhoI.mCherry.R	actgacCTCGAGcaattgaacgcatgagaaagcc
P.mCherry.F	gtccaagactagtaacgatggttg
P.PLtetO-GFP.SacI.R	atcgtaGAGCTCacgtctgtgcaagtactactgt
P.PLtetO-GFP.XbaI.F	atgctaTCTAGAcaactggcacgacaggtttc
P.cvaC.mut.F	gagaactctgactctaaatgaattagattc
P.cvaC.mut.R	gctgtttcctcctcgatg

Table 6: Fluorescence channels for the Attune NxT flow cytometer.

Excitation laser	Emission filter (nm)	Fluorescent protein
Violet (405 nm)	440/50	BFP
	512/25	CFP
Blue (488 nm)	530/30	GFP
Yellow (561 nm)	620/15	mCherry

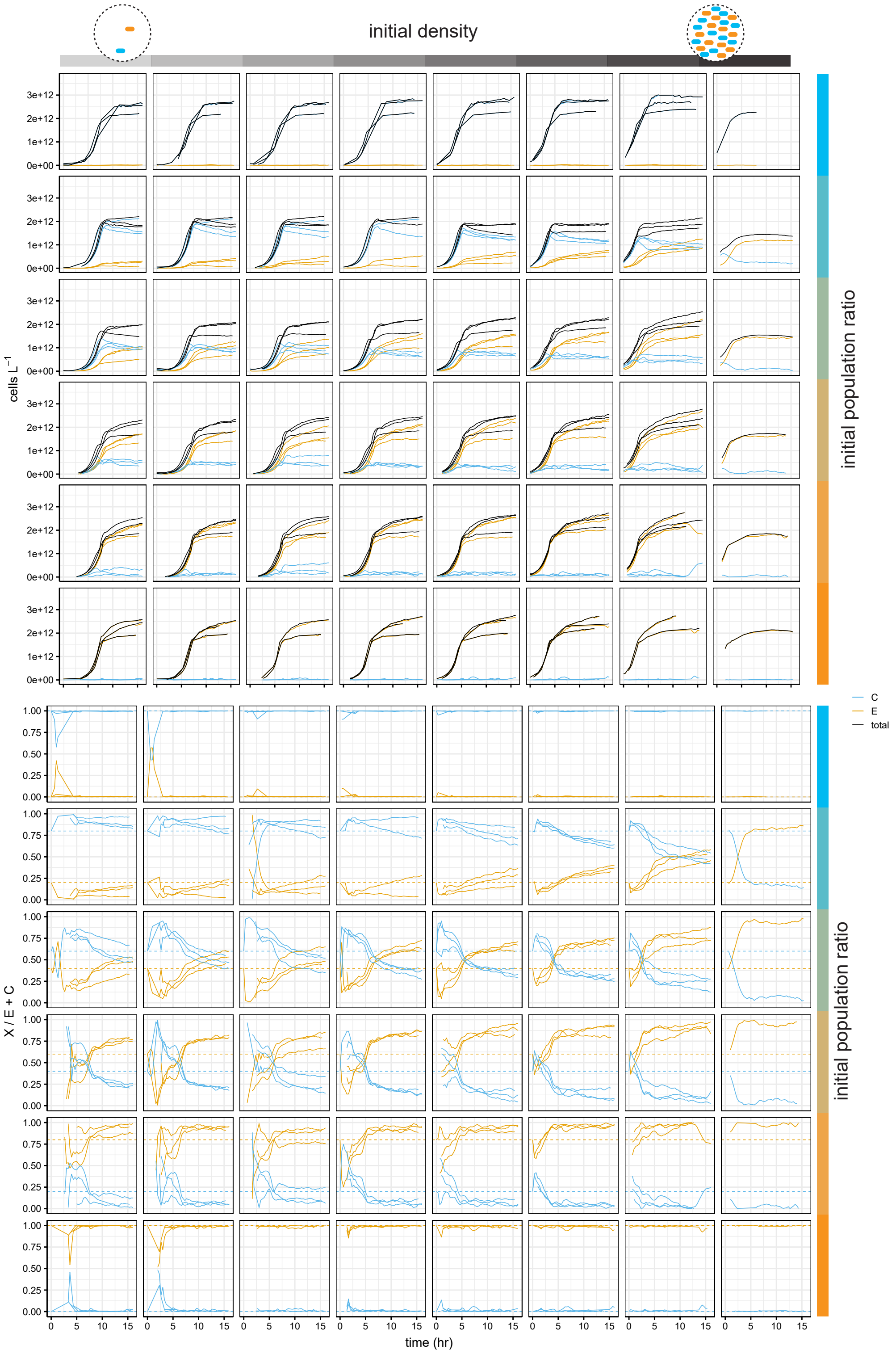

Figure 1: Competition at a variety of initial densities and population ratios. Solid lines show each of the three replicates. Data used in Figure 1G.

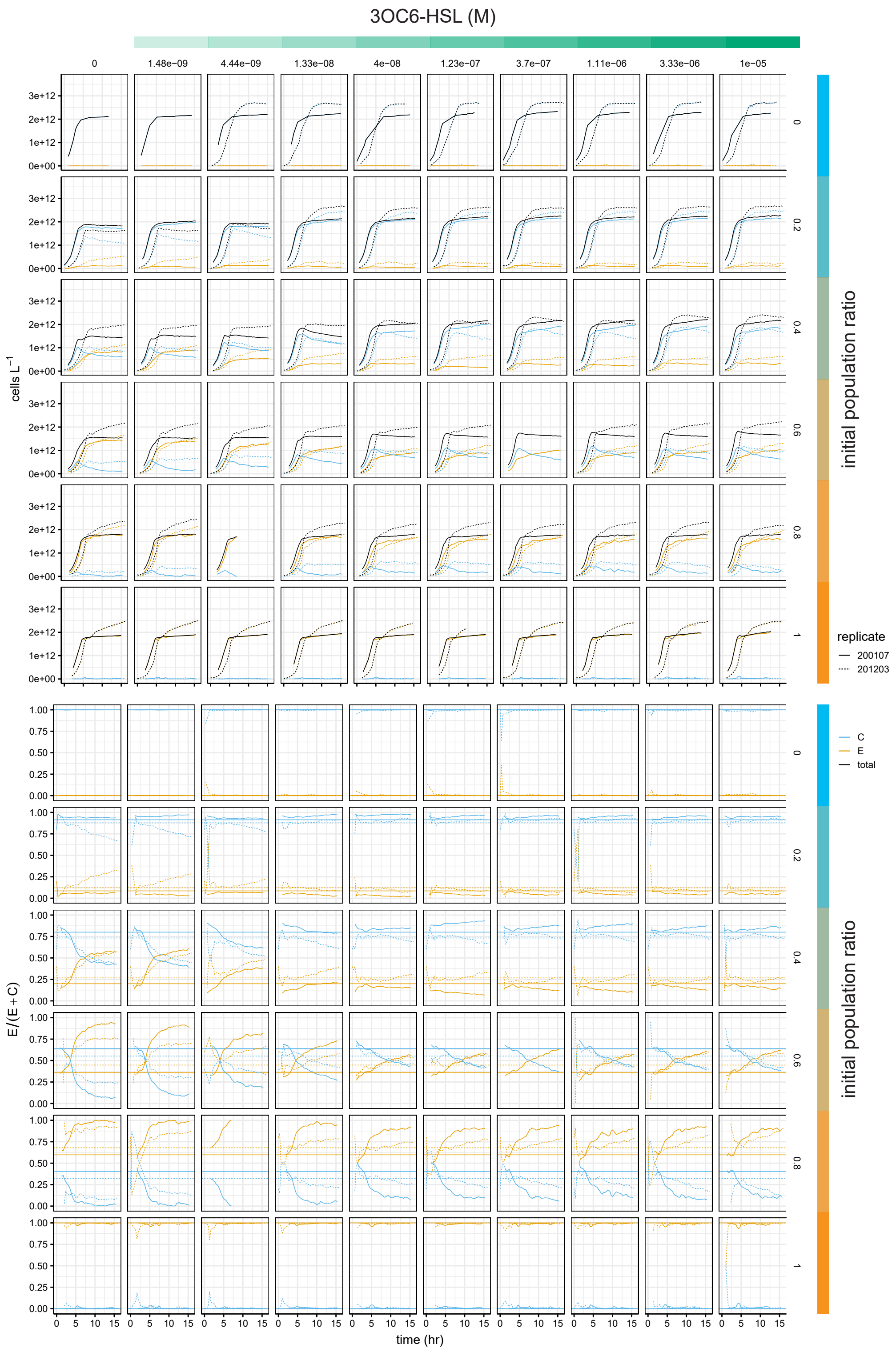

Figure 2: Competition at a variety of quorum molecule concentrations and initial population ratios. Replicate 200107 used in Figure 2F.

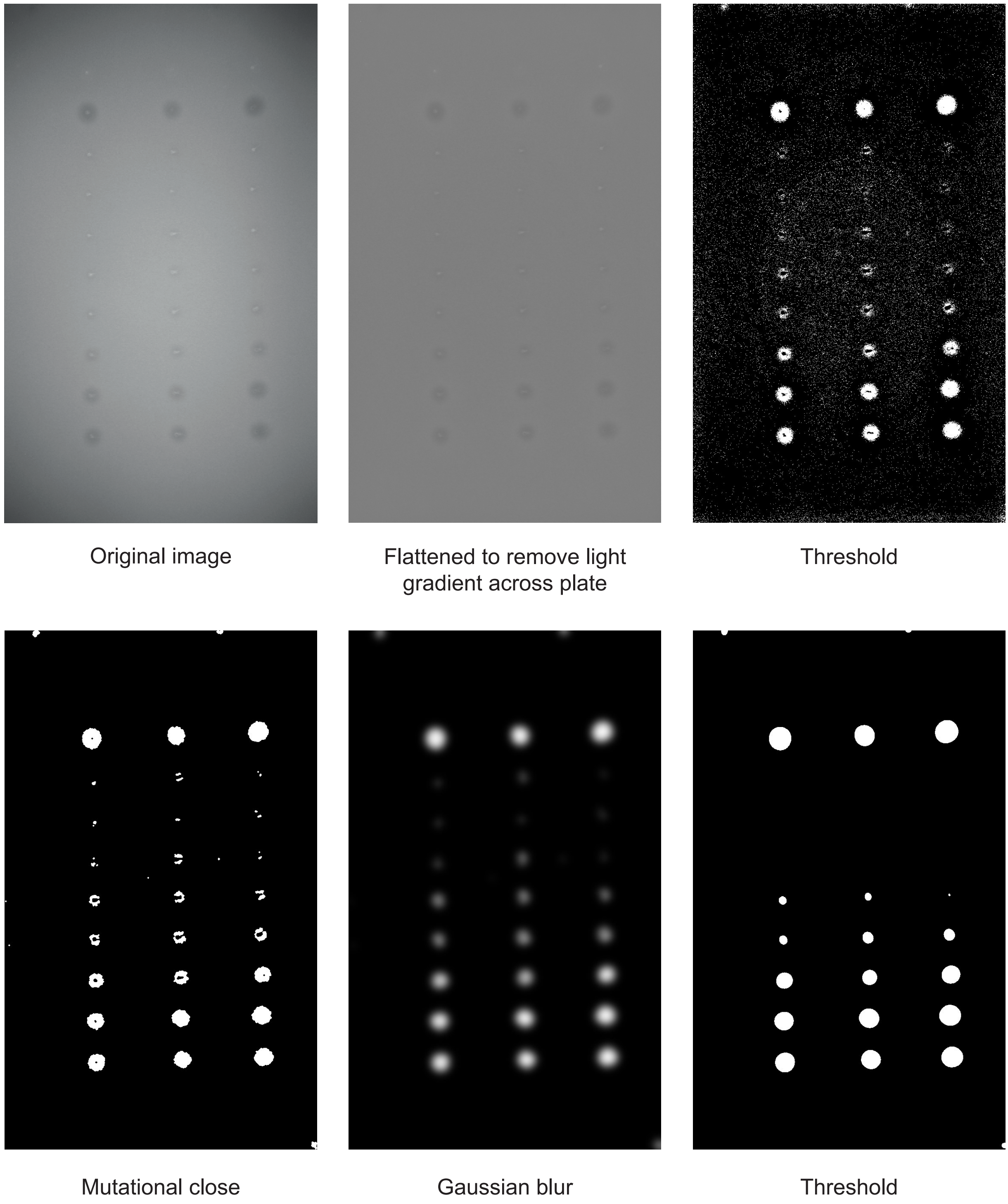

Figure 3: Image processing of agar plate spot inhibition assay.

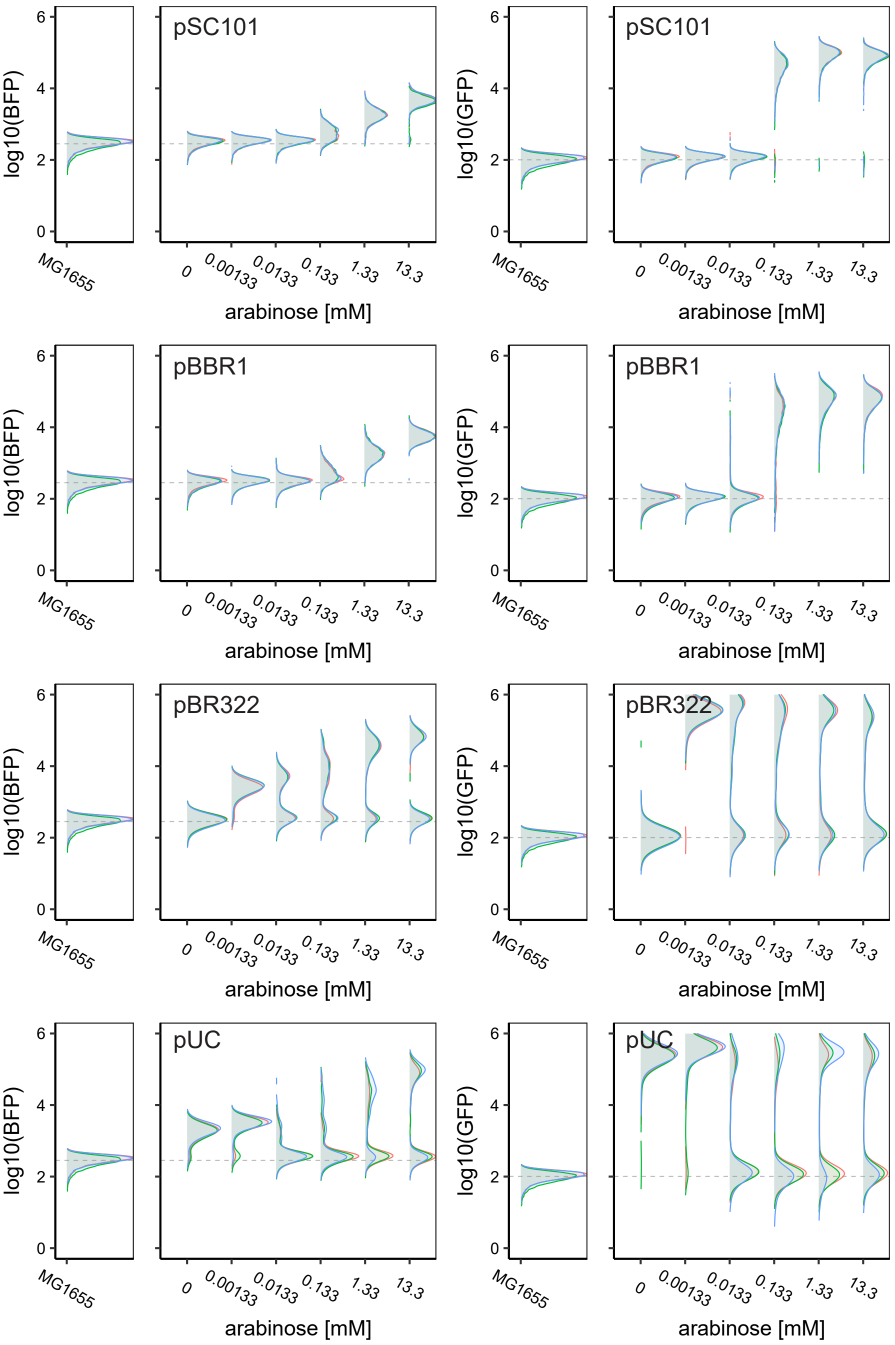

Figure 4: Changing plasmid copy number of the plasmid carrying the arabinose inducible LuxI (BFP) and 3OC6-HSL inducible TetR (GFP).

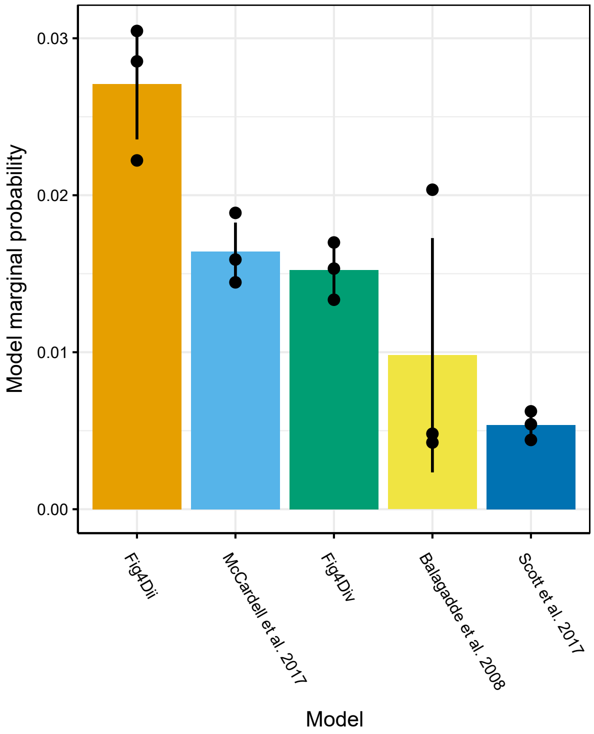

Figure 5: Comparison of model marginal probabilities, with a coexistence objective, for our system relative to previously described systems. Each bar shows the mean +/- standard deviation, of three replicates, each containing 18,000 particles. Black points show the outcome of each replicate.

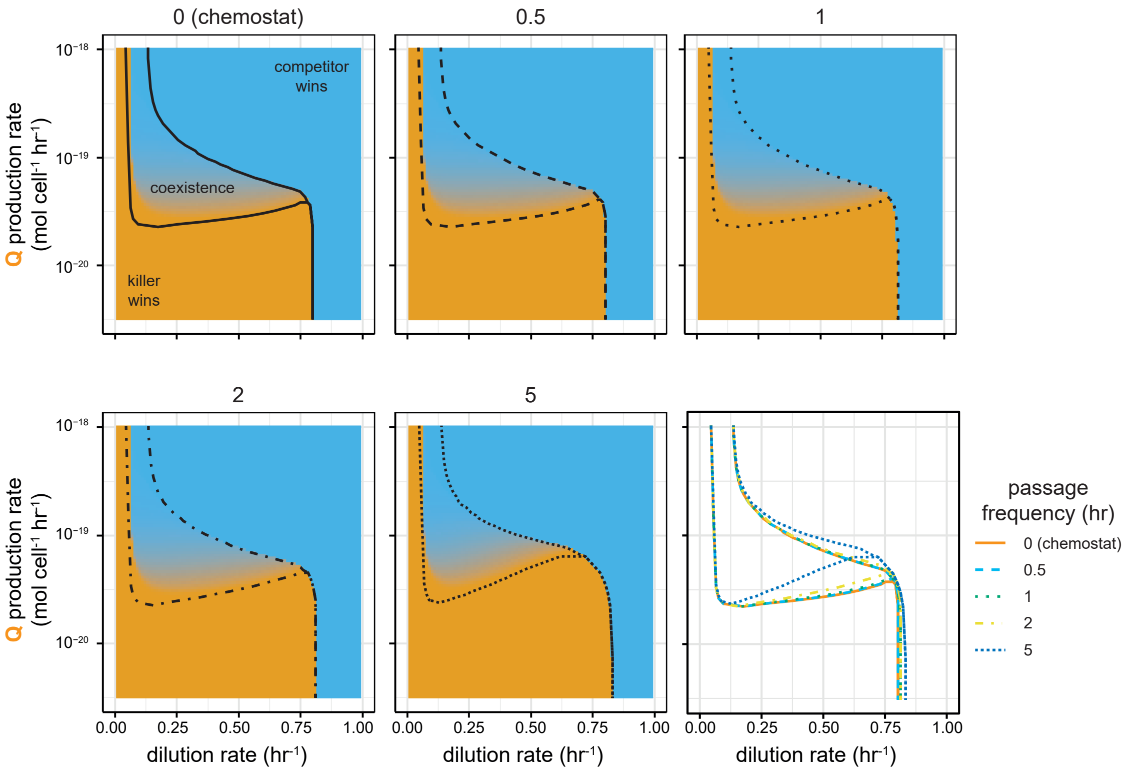

Figure 6: Comparison of "steady" states achieved by chemostat and repeated batch by computational simulation. The mathematical model was simulated for 1000 hours and the final co-culture ratios were calculated. A passage frequency of 2 hours or less provides a close approximation to the constant dilution of a chemostat.

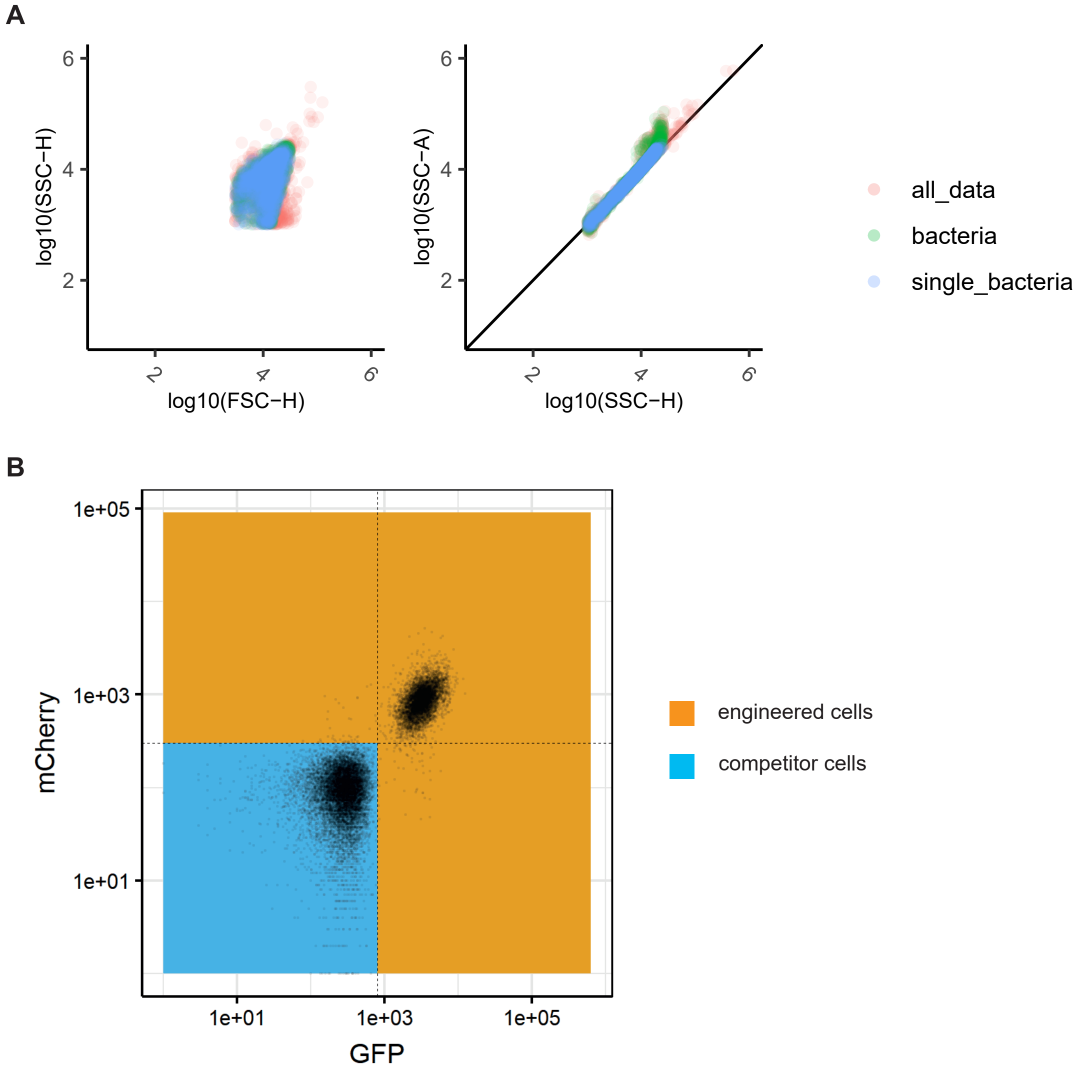

Figure 7: (A) Trimming of flow cytometry data to select only singlet bacterial events is performed by flopR [10]. (B) Clustering of engineered and competitor strains from flow cytometry data. Competitor strain events are classified as those events falling below a GFP and mCherry threshold. Engineered events are those events in which at least one of GFP and mCherry are above the thresholds. Thresholds were identical for all samples and were based on measurements of negative and positive controls.

References

[1] Pauli Virtanen, Ralf Gommers, Travis E. Oliphant, Matt Haberland, Tyler Reddy, David Cournapeau, Evgeni Burovski, Pearu Peterson, Warren Weckesser, Jonathan Bright, Stéfan J. van der Walt, Matthew Brett, Joshua Wilson, K. Jarrod Millman, Nikolay Mayorov, Andrew R. J. Nelson, Eric Jones, Robert Kern, Eric Larson, C. J. Carey, İlhan Polat, Yu Feng, Eric W. Moore, Jake VanderPlas, Denis Laxalde, Josef Perktold, Robert Cimrman, Ian Henriksen, E. A. Quintero, Charles R. Harris, Anne M. Archibald, Antônio H. Ribeiro, Fabian Pedregosa, and Paul van Mulbregt. SciPy 1.0: fundamental algorithms for scientific computing in Python. *Nature Methods*, 17(3):261–272, mar 2020.

[2] Behzad D. Karkaria, Alex J. H. Fedorec, and Chris P. Barnes. Automated design of synthetic microbial communities. *Nature Communications*, 12(1):672, dec 2021.

[3] Tina Toni, David Welch, Natalja Strelkowa, Andreas Ipsen, and Michael P.H Stumpf. Approximate Bayesian computation scheme for parameter inference and model selection in dynamical systems. *Journal of The Royal Society Interface*, 6(31):187–202, feb 2009.

[4] Oksana M. Subach, Paula J. Cranfill, Michael W. Davidson, and Vladislav V. Verkhusha. An Enhanced Monomeric Blue Fluorescent Protein with the High Chemical Stability of the Chromophore. *PLoS ONE*, 6(12):e28674, dec 2011.

[5] Arthur Prindle, Phillip Samayoa, Ivan Razinkov, Tal Danino, Lev S Tsimring, and Jeff Hasty. A sensing array of radically coupled genetic 'biopixels'. *Nature*, 481(7379):39–44, jan 2012.

[6] Kevin D Litcofsky, Raffi B Afeyan, Russell J Krom, Ahmad S Khalil, and James J Collins. Iterative plug-and-play methodology for constructing and modifying synthetic gene networks. *Nature Methods*, 9(11):1077–1080, oct 2012.

[7] Kathryn Geldart, Brittany Forkus, Evelyn McChesney, Madeline McCue, and Yiannis Kaznessis. pMPES: A Modular Peptide Expression System for the Delivery of Antimicrobial Peptides to the Site of Gastrointestinal Infections Using Probiotics. *Pharmaceuticals*, 9(4):60, oct 2016.

[8] Kyoung-Hee Choi, Jared B Gaynor, Kimberly G White, Carolina Lopez, Catharine M Bosio, RoxAnn R Karkhoff-Schweizer, and Herbert P Schweizer. A Tn7-based broad-range bacterial cloning and expression system. *Nature Methods*, 2(6):443–448, jun 2005.

[9] Tomoya Baba, Takeshi Ara, Miki Hasegawa, Yuki Takai, Yoshiko Okumura, Miki Baba, Kirill A. Datsenko, Masaru Tomita, Barry L. Wanner, and Hirotada Mori. Construction of Escherichia coli K-12 in-frame, single-gene knockout mutants: the Keio collection. *Molecular Systems Biology*, 2(1), jan 2006.

[10] Alex J. H. Fedorec, Clare M. Robinson, Ke Yan Wen, and Chris P. Barnes. FlopR: An Open Source Software Package for Calibration and Normalization of Plate Reader and Flow Cytometry Data. *ACS Synthetic Biology*, page acssynbio.0c00296, sep 2020.
