# Supplementary material for: Single strain control of microbial consortia
Source: Nat Commun. 2021 Mar 30;12:1977. doi: 10.1038/s41467-021-22240-x (PMC8010080; doi:10.1038/s41467-021-22240-x)
Supplement: Supplementary file 2 — Reporting Summary [file 41467_2021_22240_MOESM2_ESM.pdf]

nature research

Corresponding author(s): Christopher Barnes

Last updated by author(s): Feb 22, 2021

Reporting Summary

Nature Research wishes to improve the reproducibility of the work that we publish. This form provides structure for consistency and transparency in reporting. For further information on Nature Research policies, see our Editorial Policies and the Editorial Policy Checklist.

Statistics

For all statistical analyses, confirm that the following items are present in the figure legend, table legend, main text, or Methods section.

n/a	Confirmed	
☐	☒	The exact sample size (n) for each experimental group/condition, given as a discrete number and unit of measurement
☐	☒	A statement on whether measurements were taken from distinct samples or whether the same sample was measured repeatedly
☒	☐	The statistical test(s) used AND whether they are one- or two-sided<br>*Only common tests should be described solely by name; describe more complex techniques in the Methods section.*
☒	☐	A description of all covariates tested
☐	☒	A description of any assumptions or corrections, such as tests of normality and adjustment for multiple comparisons
☐	☒	A full description of the statistical parameters including central tendency (e.g. means) or other basic estimates (e.g. regression coefficient) AND variation (e.g. standard deviation) or associated estimates of uncertainty (e.g. confidence intervals)
☒	☐	For null hypothesis testing, the test statistic (e.g. F, t, r) with confidence intervals, effect sizes, degrees of freedom and P value noted<br>*Give P values as exact values whenever suitable.*
☐	☒	For Bayesian analysis, information on the choice of priors and Markov chain Monte Carlo settings
☒	☐	For hierarchical and complex designs, identification of the appropriate level for tests and full reporting of outcomes
☒	☐	Estimates of effect sizes (e.g. Cohen's d, Pearson's r), indicating how they were calculated

Our web collection on statistics for biologists contains articles on many of the points above.

Software and code

Policy information about availability of computer code

Data collection	- Plate reader data was collected using Tecan Spark Control.<br>- Flow cytometry data was collected using Attune NxT Software.<br>- Agar plate images were captured on a UVP GelDoc-It imager with GraphicsMagick 1.3.7 software.<br>- Mathematical models were simulated in Python 3 using SciPy 1.4.0.<br>- Model space exploration data was generated using AutoCD (Karkaria et al., 2021) (https://github.com/ucl-cssb/autocd).
Data analysis	- Plate reader and flow cytometry data was processed using FlopR 0.3.0 (Fedorec et al., 2020) (https://github.com/ucl-cssb/flopr)<br>- Flow cytometry competition data was clustered using custom R scripts (R >= 3.5.3, flowCore 1.52.1, dplyr >= 0.8.0)<br>- Agar plate images were processed using Adobe Photoshop CC 2019 and Fiji (March 2019) with Auto Threshold v1.17.2<br>- Bayesian model fitting was performed using R >= 3.5.3, RStan >= 2.19.2<br>- All data was plotted using R >= 3.5.3, ggplot2 >= 3.1.1, dplyr >= 0.8.0

For manuscripts utilizing custom algorithms or software that are central to the research but not yet described in published literature, software must be made available to editors and reviewers. We strongly encourage code deposition in a community repository (e.g. GitHub). See the Nature Research guidelines for submitting code & software for further information.

Data

Policy information about availability of data

All manuscripts must include a data availability statement. This statement should provide the following information, where applicable:

- Accession codes, unique identifiers, or web links for publicly available datasets
- A list of figures that have associated raw data
- A description of any restrictions on data availability

All data, including plasmid maps, are available at https://doi.org/10.5281/zenodo.4421105.

Field-specific reporting

Please select the one below that is the best fit for your research. If you are not sure, read the appropriate sections before making your selection.

[x] Life sciences [ ] Behavioural & social sciences [ ] Ecological, evolutionary & environmental sciences

For a reference copy of the document with all sections, see nature.com/documents/nr-reporting-summary-flat.pdf

Life sciences study design

All studies must disclose on these points even when the disclosure is negative.

Sample size	- ABC SMC population sizes were chosen depending on what could feasibly be completed during an allocated HPC clock time.<br>- Sample sizes for wetlab experiments were chosen to maximise the number of data points given the restrictions of the equipment used i.e. number of wells in a microtitre plate.
Data exclusions	Blank media wells on microtitre plates were excluded if they showed growth. Specifically 5 of 20 blank wells for dilution experiments and 1 of 24 blank wells for the AHL concentration experiments.
Replication	- ABC SMC replicates were aggregated and split into three groups. Standard deviation between groups was used to confirm adequate representation of the posterior distributions.<br>- Flow cytometry characterisations and agar spot inhibition assays were performed in triplicate.<br>- Plate reader experiments were performed with between 2 and 4 replicates depending on the experimental conditions. All replicates were successful and are plotted as individual data points.
Randomization	- Prior distributions were sampled from randomly.<br>- Isogenic bacteria were used so no randomization was required to control for genomic differences.
Blinding	Blinding does not apply to our study. Data processing was automated.

Reporting for specific materials, systems and methods

We require information from authors about some types of materials, experimental systems and methods used in many studies. Here, indicate whether each material, system or method listed is relevant to your study. If you are not sure if a list item applies to your research, read the appropriate section before selecting a response.

Materials & experimental systems

n/a	Involved in the study	
[x]	[ ]	Antibodies
[x]	[ ]	Eukaryotic cell lines
[x]	[ ]	Palaeontology and archaeology
[x]	[ ]	Animals and other organisms
[x]	[ ]	Human research participants
[x]	[ ]	Clinical data
[x]	[ ]	Dual use research of concern

Methods

n/a	Involved in the study	
[x]	[ ]	ChIP-seq
[ ]	[x]	Flow cytometry
[x]	[ ]	MRI-based neuroimaging

Flow Cytometry

Plots

Confirm that:

☒ The axis labels state the marker and fluorochrome used (e.g. CD4-FITC).

☒ The axis scales are clearly visible. Include numbers along axes only for bottom left plot of group (a 'group' is an analysis of identical markers).

☒ All plots are contour plots with outliers or pseudocolor plots.

☒ A numerical value for number of cells or percentage (with statistics) is provided.

Methodology

Sample preparation	Bacterial cultures were diluted 1:200 into sterile PBS.
Instrument	Attune NxT flow cytometer with autosampler
Software	Data was collected using the Attune NxT Software version 2.6. Data was processed using FlopR (https://github.com/ucl-cssb/flopr) and analysed using custom R scripts.
Cell population abundance	All events were recorded with a minimum of 10000 events falling within a "singlet" gate.
Gating strategy	Gating was performed by FlopR (as describe in Fedorec et al 2020 ACS Syn Biol) using a mixture model on FSC-H vs SSC-H to remove background debris and a linear model on SSC-H vs SSC-A to remove doublets. Classification was performed using a threshold on GFP and mCherry, with the values determined from negative and positive controls (as shown in the Supplementary Information)

☒ Tick this box to confirm that a figure exemplifying the gating strategy is provided in the Supplementary Information.
